# Quantitative and qualitative evaluation of the hippocampal cytoarchitecture in adult cats with regard to the pathological diagnosis of hippocampal sclerosis

**DOI:** 10.1371/journal.pone.0268010

**Published:** 2022-05-13

**Authors:** Jessica Zilli, Anne Schänzer, Kathrin Büttner, Monika Kressin, Martin J. Schmidt

**Affiliations:** 1 Department of Veterinary Clinical Sciences, Small Animal Clinic, Justus-Liebig-University, Giessen, Hessen, Germany; 2 Institute of Neuropathology, Justus-Liebig-University, Giessen, Hessen, Germany; 3 Institute for Biomathematics, Justus-Liebig-University, Giessen, Hessen, Germany; 4 Institute for Veterinary Anatomy, Histology and Embryology, Justus-Liebig-University, Giessen, Hessen, Germany; Nathan S Kline Institute, UNITED STATES

## Abstract

Cats are known to be affected by hippocampal sclerosis, potentially causing antiseizure drug(s) resistance. In order to lay the foundation for a standardized, systematic classification and diagnosis of this pathology in cats, this prospective study aimed at evaluating normal reference values of cellular densities and the cytoarchitecture of the feline hippocampus. Three transverse sections (head, body and tail) of each left hippocampus were obtained from 17 non-epileptic cats of different brachycephalic and mesocephalic breeds and age classes (range: 3–17 years). Histological (hematoxylin and eosin, Nissl) and immunohistochemical (NeuN, GFAP) staining was performed to investigate neuron and astroglial cell populations, as well as the layer thickness of the pyramidal cell layer and granule cell layer. Significant differences in neuronal density (in CA2-CA4 and the granule cell layer) and layer thickness (in CA1-CA3 and the granule cell layer) were evidenced throughout the longitudinal hippocampal axis (p<0.05); on the other hand, the astrocyte density did not differ. Moreover, reference ranges were defined for these parameters in the pyramidal cell layer and in the granule cell layer. The findings did not differ according to breed or age. In veterinary medicine these parameters have not been evaluated in cats so far. As surgical treatment may become a therapeutic option for cats with temporal lobe epilepsy, estimating normal values of the hippocampal cytoarchitecture will help in the standardized histopathological examination of resected hippocampal specimens to reach a diagnosis of hippocampal sclerosis.

## Introduction

One of the most common neurological condition presented by cats is epileptic seizures. The main etiologic categories associated to this condition are reactive seizures and structural epilepsy, but idiopathic epilepsy is also diagnosed [1]. The etiology of idiopathic epilepsy in dogs is mostly considered to be genetic [2]; although, no gene defect has been identified in cats so far, a familial spontaneous epileptic feline strain is known in laboratory cats in Japan [3]. Therefore, a genetic origin can be suspected in some epileptic cats, as well. With the introduction of improved magnetic resonance imaging (MRI) techniques and laboratory diagnostic methods in veterinary medicine, a high percentage of animals that were earlier diagnosed with idiopathic epilepsy are now considered to be affected by hippocampal sclerosis (HS). In some cases, HS can be the result of limbic encephalitis [4–7]. In addition, as in humans, antibodies against voltage-gated potassium channels (VGKC) can be associated with limbic pathology in cats. These antibodies recognize synaptic proteins like LGI1, CASPR2, and contactin 2. Antibody binding leads to lymphocytic infiltration, glia activation, complement-dependent neuronal damage and consequently neuronal loss in the cornu ammonis (CA) fields and dentate gyrus to varying degrees [8]. Clinical manifestations of these pathological changes are behavioral changes and complex partial seizures with orofacial involvement (FEPSO), reminiscent of seizure semiology in humans with temporal lobe epilepsy [9].

In humans, temporal lobe resection is a standardized therapy in patients with refractory temporal lobe epilepsy and HS is the most common morphological finding in resected hippocampal samples [10]. The diagnosis of HS relies on reduced pyramidal cell density and astrogliosis [6, 11]. Semiological and pathological correlations between human and feline HS makes the cat an interesting model for the study of epileptogenesis. Neurosurgical techniques for hippocampal resection in cats may not only offer a new treatment option for drug-resistant feline temporal seizures [12, 13], but also provide the opportunity to study structural changes in a defined part of the hippocampus, which is the basis for comparative studies between cats and humans. Standardization of histologic examination is important as cellular densities might not be uniform along the hippocampal longitudinal axis or differ between cat breeds. Therefore, the purpose of this study is to evaluate feline hippocampal cell layer cellular densities and cytoarchitecture in cats of different breeds.

## Materials and methods

### Animals

This study was performed on the cadavers of seventeen cats at the Small Animal Clinic- Department for Surgery and Neurology of the Justus Liebig University in Giessen. The clinical history of all of them was known. The animals were euthanized for reasons unrelated to the study. The protocol used for euthanasia was the following: premedication with 0.5 mg/kg of diazepam administered intravenously [IV], followed by induction of anesthesia with 2–4 mg/kg of propofol [IV] and finally administration of 60 mg/kg of pentobarbital [IV]. Cadavers were eligible for the current study if they were free from clinical signs of brain disease (including seizures), evaluated through a neurologic examination before death. Animals with brain pathology at post-mortem analysis were excluded.

One additional cat with suspected idiopathic epilepsy, which was euthanized due to refractory seizures and failure of antiepileptic medication(s), was enrolled to compare the obtained reference values from normal animals with those achieved from a cat with temporal seizures.

### Classification and characteristics of the animals

The cats were divided into sex categories (male and female) and into age categories according to the feline stage guidelines provided by the American Animal Hospital Association (AAHA) and the American Association of Feline Practitioners (AAFP) [14]: prime (3–6 years) and mature (7–10 years) cats were considered together as “adult” animals and senior (11–14 years) and geriatric cats (15 years +) were considered together as “senior” animals. Moreover, breeds were subdivided into mesocephalic and brachycephalic. Since almost all animals were neutered, the influence of the entire/neutered status was not taken into consideration.

The mean age of the animals was 10 years (range 3–17 years). In one cat the exact age was not known. Four cats were female (all neutered) and 13 were male (1 entire and 12 castrated). Eight animals (100% males) were classified as adults and nine as senior (55.6% females and 44.4% males). Eleven cats were mesocephalic (8 Domestic Shorthair, 1 Domestic Longhair and 2 Maine Coon) and six were brachycephalic (3 British Shorthair, 2 Persian and 1 British Longhair). Weight ranged from 2.5–7.1 kg (median weight: 4.47 kg). Cause of death was due to different diseases (Table 1). All animals did not show any macroscopical or histological evidence of brain pathology in the examination postmortem.

**Table 1:** Clinical data of the animals included in the study.

Animals	Breed	Weight (kg)	Age	Sex	Diagnosis
1	Domestic Shorthair	4.1	13 years	Male, entire	Feline asthma
2	Domestic Shorthair	3.0	17 years	Female, neutered	Suspected nasal neoplasia
3	Domestic Shorthair	5.2	3.5 years	Male, neutered	Hypertrophic cardiomyopathy, aortic thromboembolism
4	Persian	2.5	11 years	Female, neutered	Suspected renal lymphoma
5	Domestic Shorthair	4.0	13 years	Male, neutered	Aortic thromboembolism
6	Domestic Longhair	6.1	Unknown (adult)	Male, neutered	Vertebral fracture and luxation at the level of Th 5–6, sternal luxation of the 5^th^ and 6^th^ sternebrae
7	Domestic Shorthair	3.0	14 years	Female, neutered	Chronic kidney disease
8	British Longhair	4.7	7 years	Male, neutered	Recurrent feline urinary tract disease and uroabdomen
9	British Shorthair	2.6	15 years	Male, neutered	Aortic thromboembolism
10	British Shorthair	4.8	7 years	Male, neutered	Liver insufficiency
11	Domestic Shorthair	4.7	7 years	Male, neutered	Chronic otitis externa, suspected otitis media and interna, suspected diabetes
12	Domestic Shorthair	4.4	3 years	Male, neutered	Hemolytic anemia
13	British Shorthair	7.1	13 years	Male, neutered	Septic abdomen
14	Main Coon	5.9	16 years	Male, neutered	Bladder neoplasia
15	Domestic Shorthair	3.7	10 years	Male, neutered	Aortic thromboembolism
16	Main Coon	4.7	3 years	Male, neutered	Aortic thromboembolism
17	Persian	5.6	11 years	Female, neutered	Lung edema

The epileptic animal was a 2-year-old, female neutered, Domestic Shorthair cat (weight: 4.7 kg) that was euthanized due to chronic, refractory, complex, focal epileptic fits with orofacial involvement, which did not respond adequately to antiseizure medication(s). The cat started having seizures after a stressful event (another cat was introduced in the house) and despite phenobarbital (2.5 mg/kg bid) treatment, a worsening in the frequency of the episodes was observed by the owner. Indeed, whereas at the beginning only few seizures per day occurred, within few weeks the cat worsened to the point that he was experiencing many seizures in an hour and at the end, it developed status epilepticus as well. No comorbidities were known. The owner refused to perform an MRI of the head and CSF examination ante-mortem, as well as further treatments. One day after death, the brain was removed from the skull and then processed in the same manner as described above.

### Tissue sampling and processing

**Fig 1:**
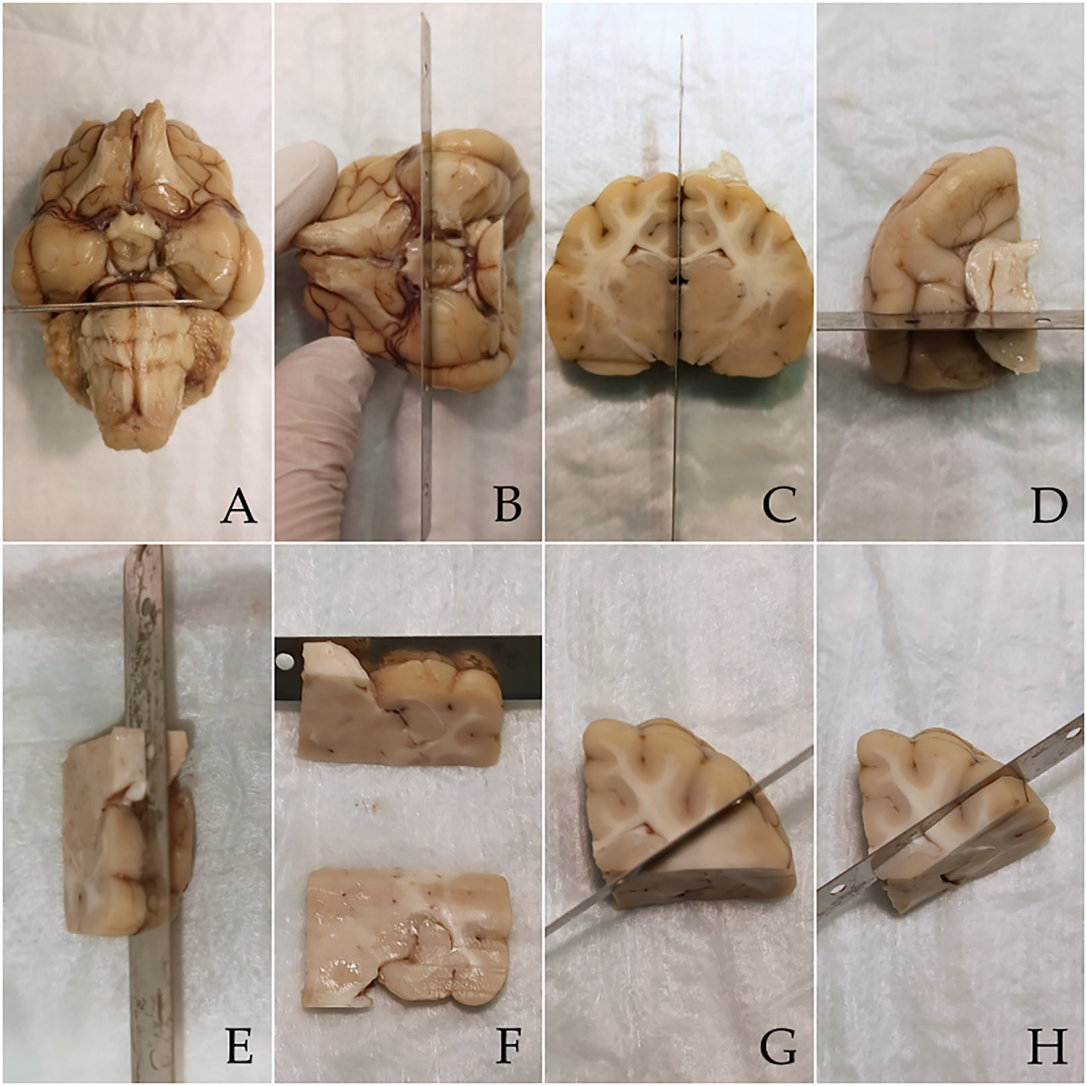
Standardized brain processing for obtaining head, body and tail sections from a hippocampus. First, the brainstem was approached ventrally and a ventro-dorsal section at the level of the rostral cerebellar colliculi and pons was trimmed with a scalpel blade in order to separate the cerebellum and medulla oblongata from the region of interest (A). Then, a transverse cut at the level of the pituitary gland was obtained with a long blade (B). Subsequently, the right and left hemispheres were separated over the midline with the same blade (C). At this point, the left hippocampal body was dissected through a 90° cut directly underneath the mesocephalic aqueduct (D). Two further parallel sections, respectively 2–4 mm under (E) and 2–4 mm over (F) the previous cut were made, in order to obtain a specimen from the head and one from the body of the hippocampus, respectively. Finally, from the residual dorsal part of the temporal lobe a section from the hippocampal tail was obtained: the cut line was set through the vertex of the occipitotemporal flexure with an inclination of 45° (G). Once done, a further cut parallel and dorsal to the previous one was performed, obtaining the dorsal hippocampal specimen (H).

Within 12 hours after death or euthanasia, all brains were removed from the cats’ skulls and fixed in 10% formaldehyde at least for one week before sectioning. Three 2–4 mm thick specimens were obtained from the dorsal (at the level of the hippocampal tail), middle (at the level of the hippocampal body) and ventral (at the level of the hippocampal head) part of each left temporal lobe (Fig 1), following the recommendations for systematic sampling and processing of brains from epileptic animals provided by the International Veterinary Epilepsy Task Force [15].

First, the brainstem was approached ventrally and a ventro-dorsal section at the level of the rostral cerebellar colliculi and pons was trimmed with a blade (scalpel blade no. 10) in order to separate the cerebellum and medulla oblongata from the region of interest (temporal and occipital lobes) (Fig 1A). Then, a transverse brain cut at the level of the pituitary gland was obtained with a long blade (Fig 1B). Subsequently, the right and left hemispheres were separated through a cut over the midline with a long blade (Fig 1C). For the next part, only the left hemisphere was used. At this point, the hippocampal body was dissected through a 90° cut directly underneath the mesocephalic aqueduct: the blade was inserted into the caudal surface of the rostral mesencephalic stump in a tilted caudoventral to rostrodorsal fashion (90°), to create a perpendicular section of the entorhinal cortex and temporoventral hippocampal body (Fig 1D). At this stage, two further parallel sections, respectively 2–4 mm over and 2–4 mm under the previous cut were made, in order to obtain a specimen from the body and one from the head of the hippocampus, respectively (Fig 1E and 1F). Finally, from the residual dorsal part of the temporal lobe a section from the hippocampal tail was obtained: the cut line was set through the vertex of the occipitotemporal flexure with an inclination of 45° (Fig 1G). Once done, a further cut parallel and dorsal to the previous one was performed, obtaining the dorsal hippocampal specimen (Fig 1H).

All obtained temporal lobe samples were post-fixed in 10% neutral-buffered formalin for at least a further 5 days at room temperature and paraffin embedded according to standard procedures.

### Histology and immunohistochemistry stainings

Wax blocks were sectioned with a Leica RM2125 RTS microtome^®^ (Leica Biosystems, Wetzlar, Germany) at 7 μm.

The slides were stained using hematoxylin-eosin (HE) and Nissl staining according to standard procedures. The completeness and quality of the histological sections was evaluated with the HE staining and in case the hippocampus was damaged or partly missing, the specimen was trimmed again, and new slides were prepared for the HE assessment and the other staining (Nissl, NeuN and GFAP).

In brief, to perform Nissl staining, the slides were pre-treated with a 50% potassium disulfite solution for 15–20 minutes before applying a 1.5% cresyl-violet solution for 20 minutes at room temperature.

Immunohistochemical analyses were performed using the ultraView DAB kit^®^ and the staining platform BenchMark XT ULTRA (Ventana, Heidelberg, Germany). All slides were deparaffinized through a passage in a 100% xylene solution and then hydrated. During the deparaffinization process, the slides were heated up to a temperature of 72°C to improve paraffine removal.

Before starting the NeuN staining, a pre-treatment at 95°C was performed. During this procedure, the ULTRA cell conditioning solution #1 (EDTA with pH = 9) was applied on the slides. The treatment lasted 64 minutes. Subsequently, one drop of ultraView DAB detection kit, which contains a cocktail of secondary antibodies (goat-anti-mouse-IgG, goat-anti-mouse-IgM and goat-anti-rabbit), the chromogen (3,3’ -diaminobenzidintetrahydrochlorid), hydrogen peroxide and copper sulphate, and one of prep kit 25^®^ (monoclonal mouse anti-NeuN MAB377; Merck, Darmstadt, Germany) with a concentration of 1:500 were applied on each slide and then all of them were pre-heated up to a temperature of 42°C for four minutes before incubation (32 minutes). Last, a counterstaining with hematoxylin (modified Gill´s hematoxylin) and bluing reagent (aqueous solution containing buffered lithium carbonate) was performed. The slides were then incubated for 8 minutes after staining and for 4 minutes after using the bluing reagent.

In preparation of the GFAP staining, the slides were pre-treated with 1 drop of proteases (Protease 1), to improve the immunoreactivity of the tissues. Then, they were incubated for 8 minutes. After that, one drop of ultraView DAB detection kit and one of prep kit 81^®^ (polyclonal rabbit anti-GFAP Z0334; Dako Agilent, Santa Clara, United States) were applied on the slides with a dilution of 1:2000. At this stage, the slides were incubated for 16 minutes with a temperature of 36°C. Finally, a counterstaining with hematoxylin (modified Gill´s hematoxylin) and bluing reagent was performed. A 4-minute incubation was carried out after applying each reagent.

### Histological examination

All sections were scanned with the digital slide scanner NanoZoomer S360^®^ (Hamamatsu Photonics, Herrsching am Ammersee, Germany).

**Fig 2:**
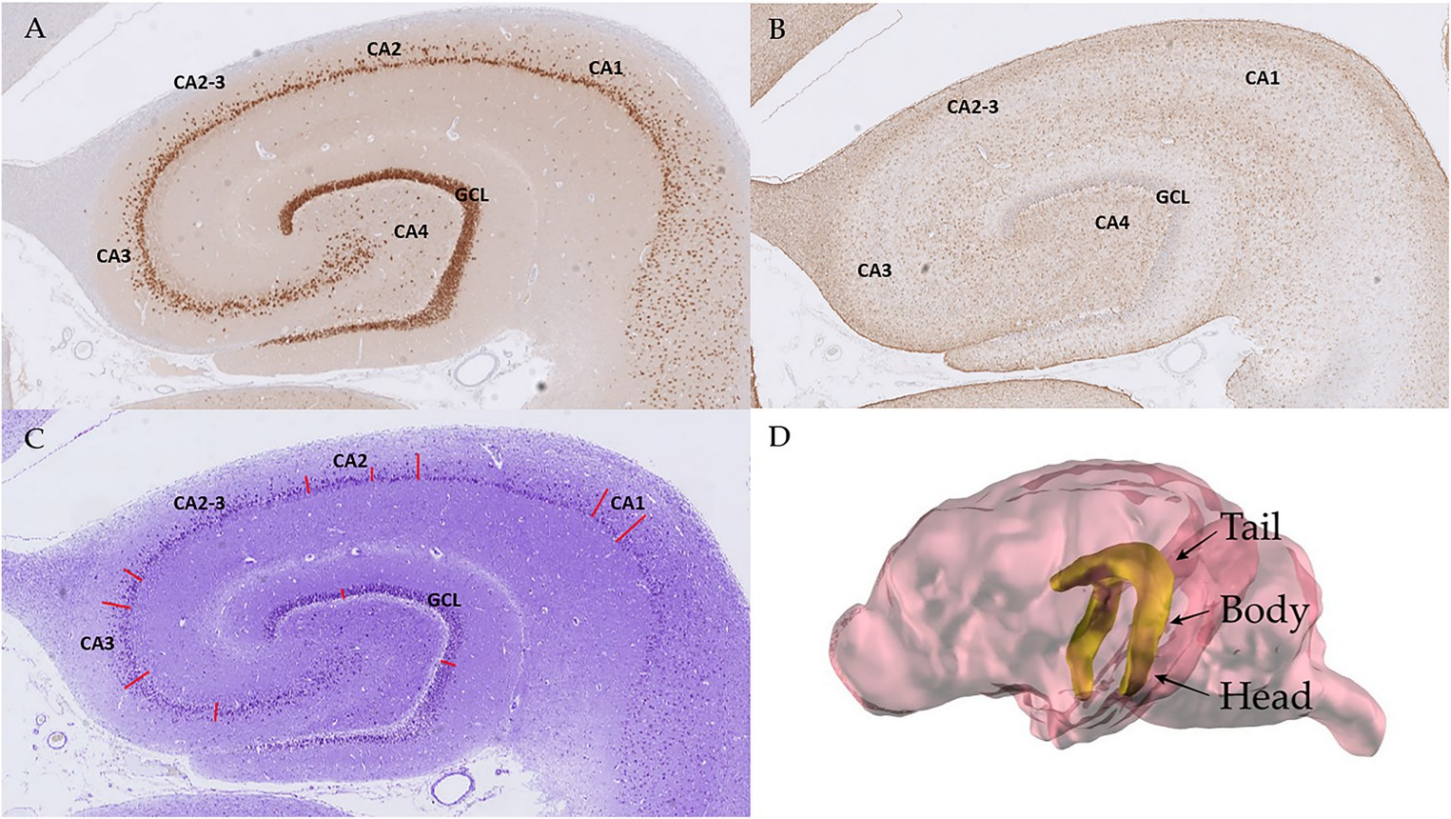
Hippocampal areas of the assessed sections. In A, B and C an example from the body of cat 17 can be observed. From each hippocampus two rectangular areas were selected from the regions CA1-4 and GCL for cell counting at NeuN (**A**) and GFAP (**B**) stained slides. CA2 alone was not assessed for GFAP. Evaluation of the LT of the different areas at Nissl-stained sections (**C**). A 3D model of the skull of a Domestic Shorthair cat based on CT and MRI images showing the localization of the hippocampus and its divisions (tail, body and head) (**D**).

Morphometric analysis of the hippocampus was performed on three sections: dorsal or tail (1), middle or body (2) and ventral or head (3). According to the feline HS assessment by Wagner et al. (2014), the cellular density and layer thickness (LT) were analyzed on the CA subfields (CA1, CA2, CA3 and CA4) and the granule cell layer (GCL) of the dentate gyrus. Since the borders of CA2 are mostly not well-defined, this area was evaluated both individually and in combination with CA3 (Fig 2).

To obtain cell densities, in each evaluated region, two rectangular areas from all CA areas of the pyramidal cell layer and from the GCL were extracted using the program QuPath-0.2.3^®^ and sent to the Fiji^®^ processing package (version 1.8; https://imagej.net/software/fiji/). The cell counter plug-in of this software was then used to run cell counts in each area. The surfaces (μm^2^) of the evaluated areas were extracted from Fiji^®^ as well and then used to calculate cellular densities (cells/mm^2^). Due to the difficulty in distinguishing the borders of CA2 with GFAP staining, astrocyte density (AD) was not evaluated in this single region, but rather by combining CA2 and CA3.

LTs were assessed by using QuPath-0.2.3^®^. For each CA region and GCL, the thickness (μm) was measured on two different, randomly selected points, in order to take account of any potential, intrinsic variability of the layers (Fig 2C). In CA4, this evaluation was not possible, due to the form of this area.

Cellular densities were evaluated both for the pyramidal cells (NeuN) and the astroglia (GFAP), and the LTs were evaluated using both Nissl and NeuN staining in order to point out any differences in the two methods.

### Statistical analysis

Statistical evaluation was performed under the supervision of a biostatistician (KB) and computed with a commercial statistical software package (SAS^®^ 9.4 Procedures Guide: Statistical Procedures, 2^nd^ edition ed., Statistical Analysis System Institute Inc., Cary, NC, USA). Data were acquired by the author and submitted in an Excel table (S1–S3 Files).

Both cellular densities and LT data were examined for normality by the Shapiro-Wilk test for each group, since one repeated measurement was conducted for every parameter in each measured area (CA1-4 and GCL) and section (tail, body and head). Then, data were evaluated via t-test for repeated measurements to determine whether any statistically significant differences between the two measurements were present. Similarly, the presence of significant differences between the LTs measured from the Nissl- or from NeuN-stained slides was assessed. If the t-test did not show any evidence of significant differences, the mean values of the repeated measurements could be then used for the further statistical assessment.

Altogether, the data were normally distributed for almost all repeated measurements. In fact, only in the evaluation of AD did the Shapiro-Wilk test show no normal distribution for the CA3 body (p = 0.03) and in the evaluation of the LT for CA1 dorsal and ventral (p = 0.04). Nevertheless, a visual inspection of the data showed that these were almost normally distributed and since the related p-values were just below 0.05, the t-test was applied for these exceptions as well.

The t-test showed that a statistically significant difference was present in some cases between the repeated measurements. This was evident in CA3 dorsal (p = 0.03), CA4 dorsal (p = 0.01) and body (p = 0.002) in the evaluation of neuronal density (ND), in CA3 ventral (p = 0.01) in the evaluation of AD and in CA3 dorsal (p = 0.048) in the evaluation of LT. Nevertheless, mean values for cellular densities and LT were used in the further analysis.

To compare cellular densities and LTs of the CA areas and GCL between the hippocampal tail (1), body (2) and head (3), a variance analysis of repeated measurements according to sections 1, 2 and 3 was performed. In this assessment, the factors age and breed were also included as variables. Sex was not included in the analysis due to the absence of female animals in the age group “adult”. The p-value for significance was set at < 0.05. Descriptive statistics were provided for estimates of cell densities and LT differences, also correlated to breed and age. A description of the quantitative data characteristics is given by mean (± SD).

Due to the deficient or absent NeuN staining in cats 5, 6 and 9, these animals were excluded from the assessment of ND. Regarding LT, while comparing the measurements from Nissl- and NeuN-stained slides, the Shapiro-Wilk test showed that all data except in CA3 ventral (p = 0.004) were normally distributed. Since almost none statistically significant differences were detected via t-test between the values obtained from NeuN or Nissl-stained slides, the LTs were only assessed from the latter, in order to evaluate the whole study population. Indeed, only in CA2-3 body were relevant differences (p = 0.015) found between the two staining methods.

## Results

### Morphometric analysis

**Fig 3:**
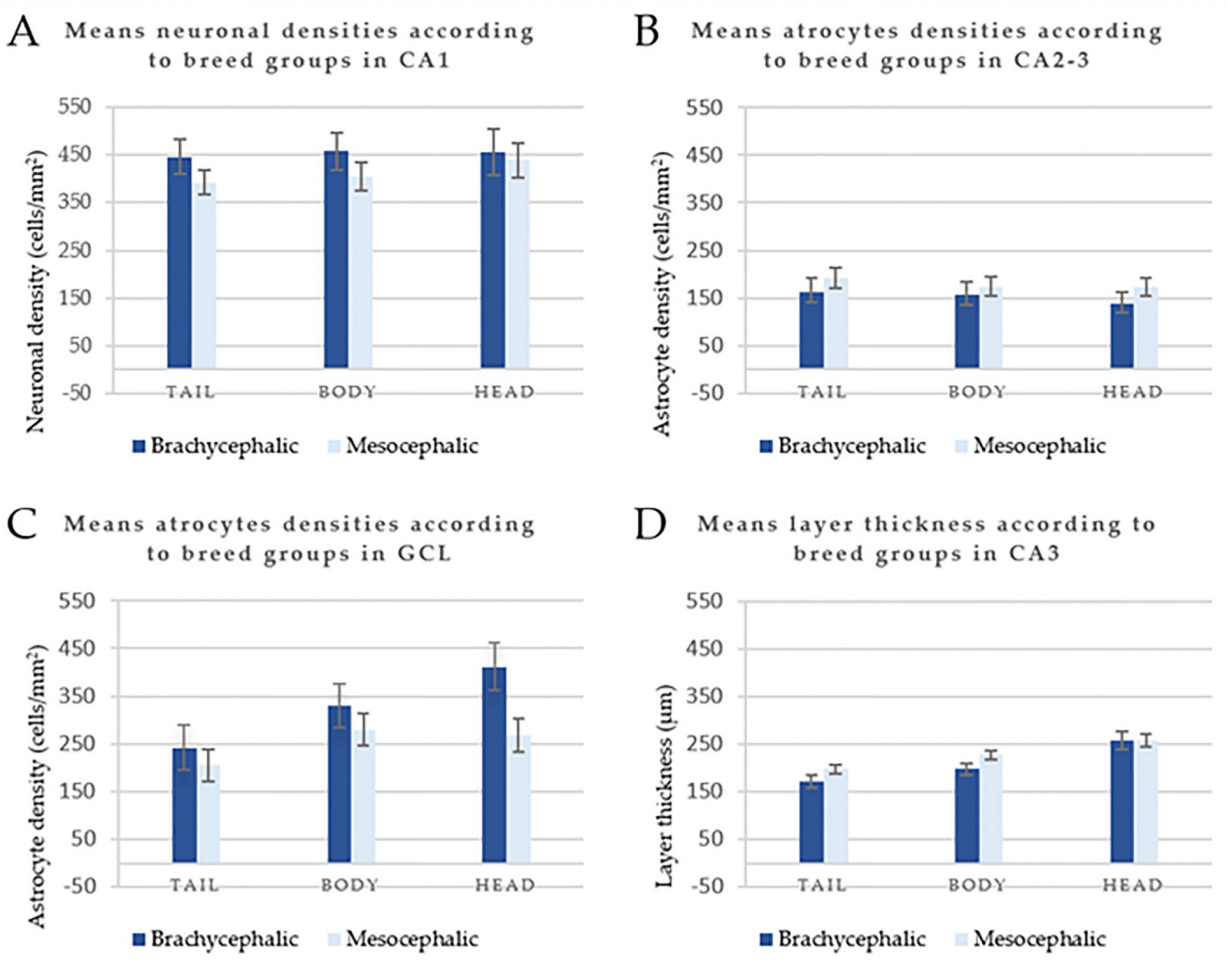
Examples of the estimated ND, AD and LT values (LS-means +/- standard error) from the variance analysis for repeated measurements comparing the hippocampal tail, body and head with the fixed effect of breed. The data are relative to neuronal densities in CA1 (A), AD in CA2-3 (B), and in the GCL (C), and LT in CA3 (D). The data were not statistically significant.

The results of the repeated measurements variance analysis revealed that age and breed did not exhibit any statistically relevant influence either on cellular densities or on LT. Nevertheless, brachycephalic cats seemed to have a higher ND than mesocephalic cats (Fig 3A). In contrast, AD were higher only in CA2-3 and CA4 in mesocephalic cats than in brachycephalic cats (Fig 3B), whereas in the GCL the opposite was observed (Fig 3C). Moreover, the hippocampal tail and body of mesocephalic cats seemed to have thicker layers (Fig 3D). However, all these results showed no statistical differences.

**Fig 4:**
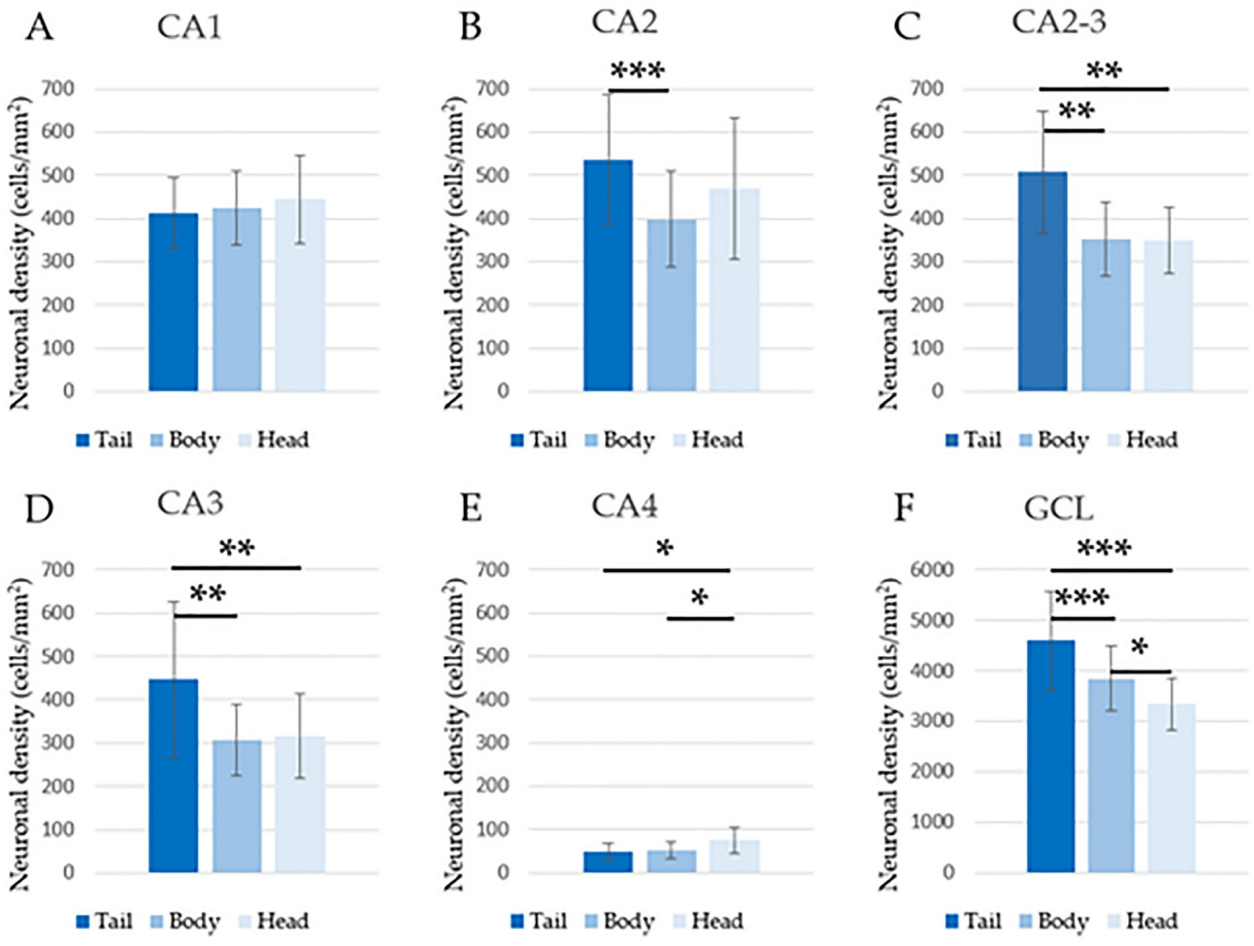
Estimated ND values (LS-means +/- standard error) (least-squares-means) from the variance analysis for repeated measurements comparing the hippocampal tail, body and head. The data are relative to all examined areas and show the presence of statistically significant differences. * p < 0.05, ** p < 0.01, *** p < 0.001.

Regarding the variance analysis of NDs evaluated in NeuN-stained slides, mean values, standard deviations and minimum and maximum values are summarized in Table 2. The analysis revealed that no statistically significant difference was present in CA1 between the head (3), body (2) and tail (1) (Fig 4A), whereas densities were significantly different between all three sections in the GCL (Fig 4F). Specifically, the values increased in the ventro-dorsal direction along the hippocampal axis. In addition, sections 1–3 had different NDs also in CA2-3, CA3 and CA4, but while density values were higher in the hippocampal tail for CA2-3 and CA3 and increased in the ventro-dorsal direction, in CA4 the NDs were higher at the level of the hippocampal head and decreased in the ventro-dorsal direction (Fig 4C–4E). Between sections 1–2 in CA2, CA2-3 and CA3, a significant difference was also evident (Fig 4B–4D). The hippocampal body and head (2–3) had significantly different densities only in CA4 and in the DG, as aforementioned, whereas sections 1–3 were significantly different in all areas apart from CA1 and CA2 (Fig 4A–4F). In comparison to the CA areas, at the level of the GCL, the standard deviations for the mean density values were overall lower. The GCL showed the highest NDs, whereas CA4 had the lowest (Table 2).

**Table 2:** Mean values, standard deviation, and minimum and maximum values for NDs.

Examined area	Hippocampal part	Numerical density (cells/mm^2^)	Maximum (cells/mm^2^)	Minimum (cells/mm^2^)
**CA1**	Tail	412.063 ± 84.315	616.395	283.32
	Body	424.45 ± 84.45	552.595	231.575
	Head	443.934 ± 101.327	601.9	239.63
**CA2**	Tail	535.915 ± 149.32	800.675	281.2
	Body	398.263 ± 110.218	564.36	210.775
	Head	468.855 ± 162.733	740.335	216.19
**CA2-3**	Tail	506.645 ± 141.282	754.95	313.21
	Body	351.408 ± 84.922	472.52	191.145
	Head	349.127 ± 77.754	482.45	249.85
**CA3**	Tail	446.513 ± 180.898	806.41	209.89
	Body	307.249 ± 81.906	470.535	189.89
	Head	315.499 ± 97.041	480.955	142.08
**CA4**	Tail	48.938 ± 18.178	76.74	7.225
	Body	52.377 ± 18.625	84.88	14.12
	Head	74.435 ± 29.667	144.265	22.405
**GCL**	Tail	4607.87 ± 975.97	6456.09	3053.81
	Body	3851.26 ± 639.91	4978.56	2851.16
	Head	3339.40 ± 523.27	4395.30	2391.16

**Fig 5:**
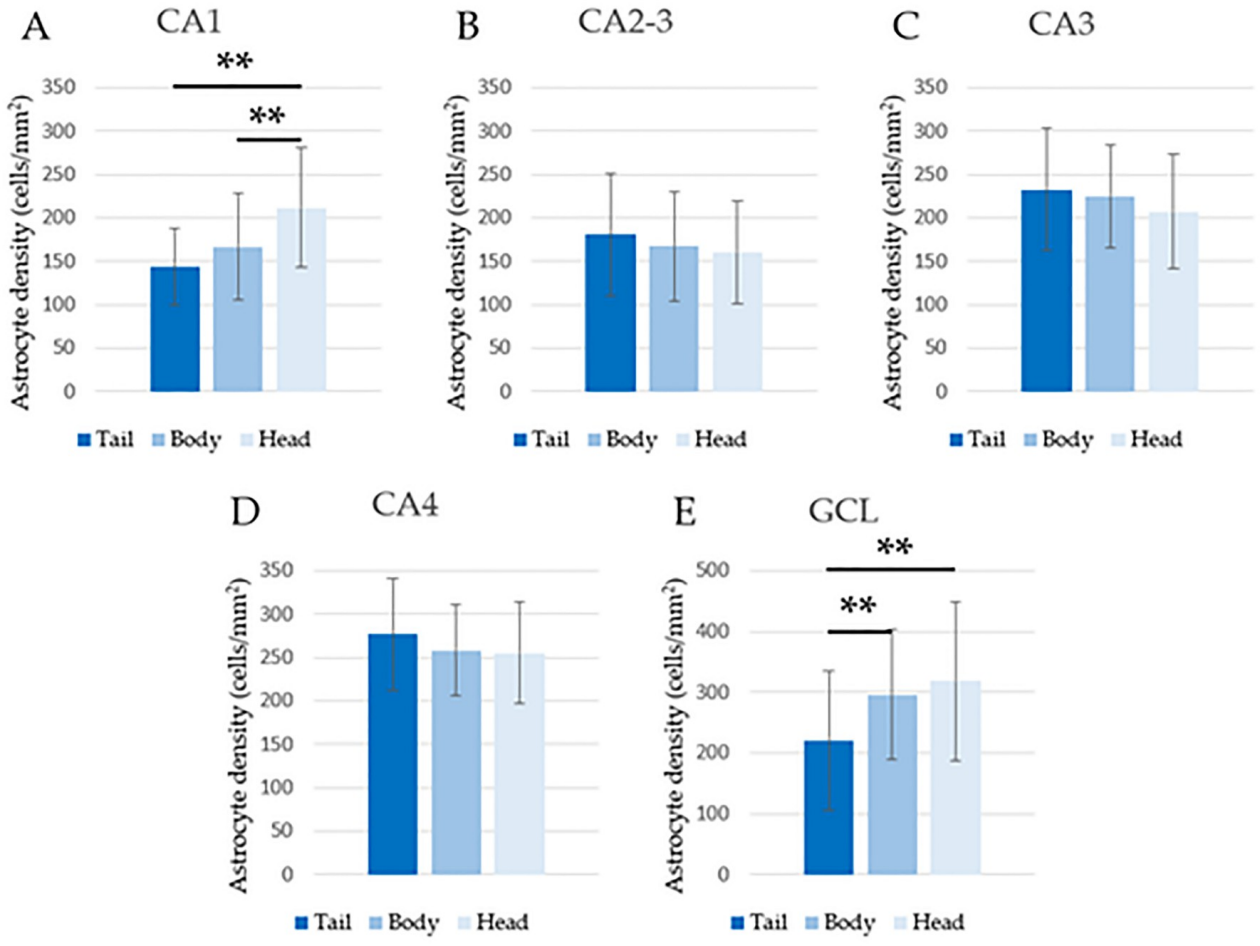
Estimated AD values (LS-means +/- standard error) from the variance analysis for repeated measurements comparing the hippocampal tail, body and head. The data are relative to all examined areas and show the presence of statistically significant differences. ** p < 0.01.

Regarding the variance analysis of ADs evaluated in GFAP-stained slides, the mean values, standard deviations and minimum and maximum values are summarized in Table 3. The AD values were statistically significantly different only between the tail and head (1–3) in CA1, and in the GCL, between the body and head (2–3) in CA1 and between the body and tail (1–2) in the GCL (Fig 5A and 5E). In all remaining hippocampal areas, the astroglial population did not show quantitatively significant differences between 1, 2 and 3 (Fig 5B–5D). In CA1, ADs were significantly higher at the level of the head (3). In the GCL, the density values were higher in the head (3) if compared to the tail (1) and in the body (2) in comparison to the tail (1) (Table 3).

**Table 3:** Mean values, standard deviation, and minimum and maximum values for ADs.

Examined area	Hippocampal part	Numerical density (cells/mm^2^)	Maximum (cells/mm^2^)	Minimum (cells/mm^2^)
**CA1**	Tail	143.497 ± 44.836	219.183	64.932
	Body	166.691 ± 61.582	283.739	68.189
	Head	211.511 ± 69.282	324.696	98.421
**CA2-3**	Tail	181.222 ± 70.51	293.408	88.173
	Body	167.334 ± 62.989	279.977	45.295
	Head	160.372 ± 59.044	301.743	90.268
**CA3**	Tail	232.54 ± 70.708	361.24	117.255
	Body	224.644 ± 59.227	330.153	112.167
	Head	207.268 ± 65.739	399.164	113.612
**CA4**	Tail	276.645 ± 64.096	368.236	125.291
	Body	258.18 ± 52.31	346.269	176.853
	Head	254.963 ± 58.29	370.259	154.576
**GCL**	Tail	220.544 ± 114.609	504.189	63.209
	Body	296.108 ± 107.069	488.89	170.81
	Head	318.379 ± 130.745	554.902	95.553

**Fig 6:**
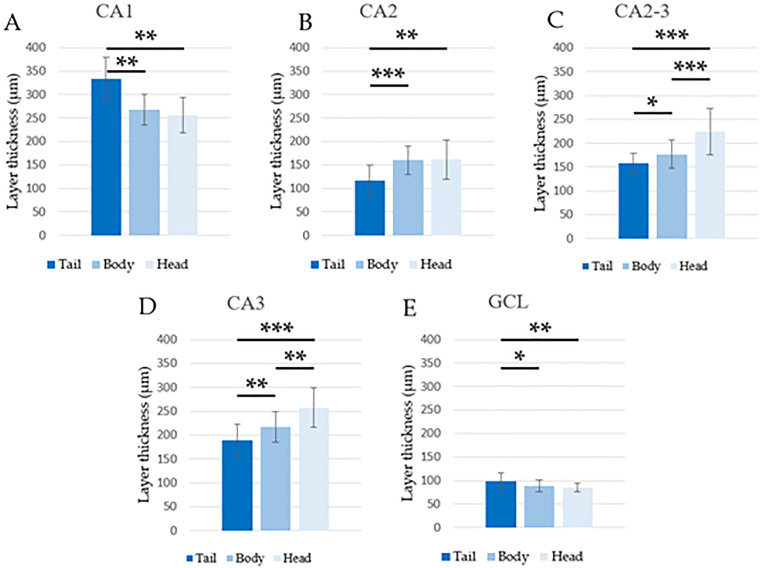
Estimated LT values (LS-means +/- standard error) from the variance analysis for repeated measurements comparing the hippocampal tail, body and head. The data are relative to all examined areas and show the presence of statistically significant differences. * p < 0.05, ** p < 0.01, *** p < 0.001.

The LTs evaluated with Nissl staining showed significant differences in almost all regions. Mean values, standard deviations and minimum and maximum values for LTs are summarized in Table 4. Between the body and head (2–3) at the level of CA1, CA2 and GCL, no difference was found (Fig 6A, 6B and 6E). In CA1 and in the GCL the LT values were larger in the tail (1) than in both the body (2) and the head (3) (Fig 6A and 6E). In general, LTs seemed to decrease in the ventro-dorsal direction at the level of CA2, CA2-3 and CA3 (Fig 6B–6D). In addition, despite the significant differences observed in the GCL, the LTs appeared more homogenous than in the CA areas (Table 4).

**Table 4:** Mean values, standard deviation, and minimum and maximum values for LT.

Examined area	Hippocampal part	Numerical density (μm)	Maximum (μm)	Minimum (μm)
**CA1**	Tail	332.791 ± 46.388	412.575	254.73
	Body	267.93 ± 33.121	315.81	211.135
	Head	256.658 ± 38.004	344.42	209.585
**CA2**	Tail	117.123 ± 32.387	202.385	72.97
	Body	159.817 ± 29.938	210.54	113.21
	Head	161.558 ± 42.139	236.595	105.075
**CA2-3**	Tail	157.414 ± 20.983	188.015	115.965
	Body	177.084 ± 30.2	238.75	139.84
	Head	224.334 ± 48.062	348.77	156.44
**CA3**	Tail	188.458 ± 34.344	258.795	119.715
	Body	217.284 ± 32.616	266.58	159.13
	Head	257.283 ± 41.512	320.12	183.71
**GCL**	Tail	98.686 ± 17.879	129.505	68.04
	Body	88.169 ± 12.139	108.725	65.965
	Head	84.978 ± 9.736	96.355	64.155

### Hippocampal pyramidal cell layer and granule cell layer cytoarchitecture in cats

**Fig 7:**
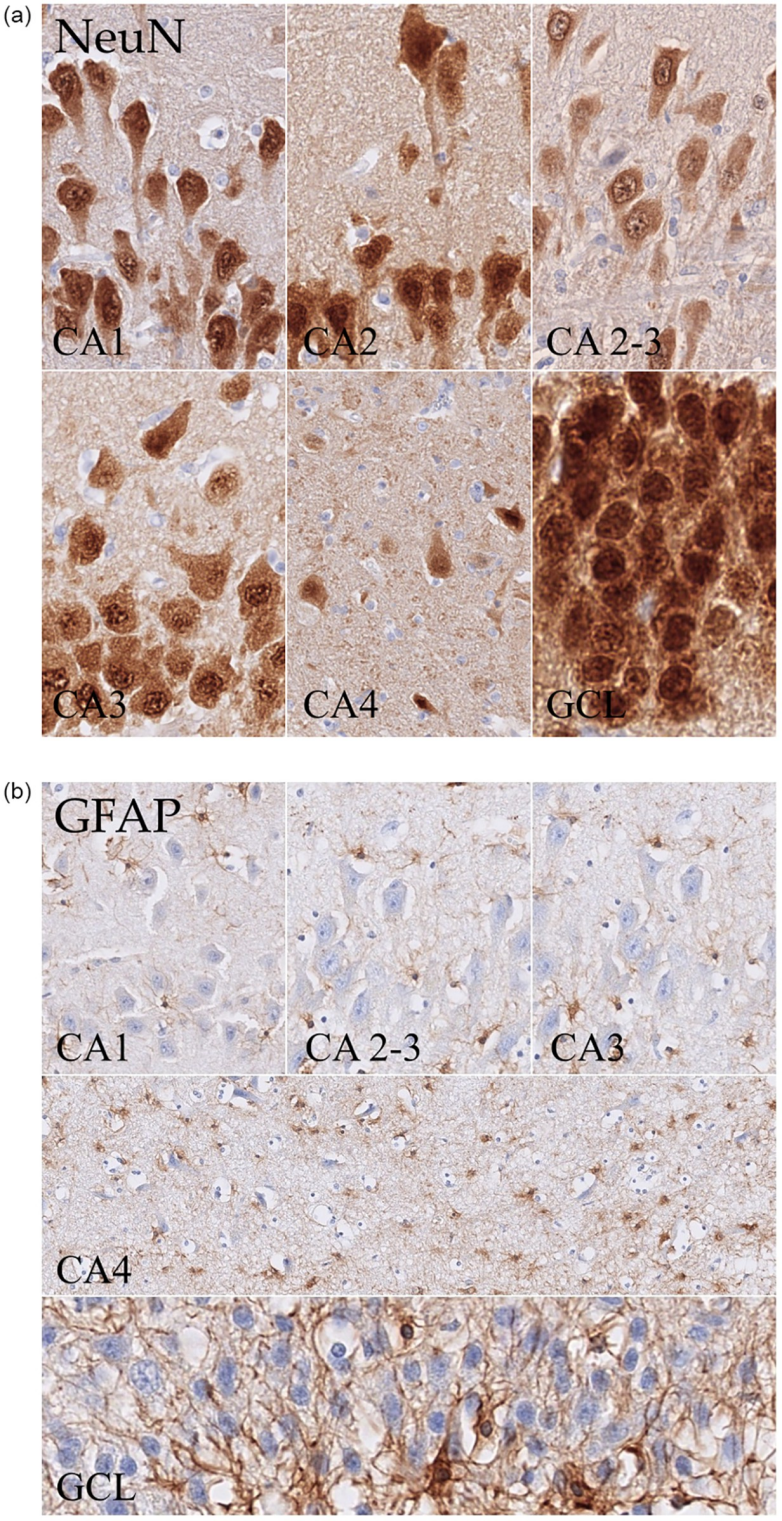
(a) (NeuN), neuronal cell morphology of the different hippocampal pyramidal layer areas and GCL. All examined areas, including the four CA regions (CA1-4), the GCL and an example of the combination of CA2 and 3 can be seen. Significant morphological differences were present along the pyramidal cell layer. Indeed, both cell form and distribution meaningfully changed along the transverse hippocampal axis. (b) (GFAP), astroglial cell morphology of the different hippocampal pyramidal layers and GCL. All examined areas, including CA1, CA3 and CA4, the GCL and an example of the combination of CA2 and 3 can be seen. The distribution of astroglia was homogenous throughout the pyramidal cell layer. Nevertheless, in CA4, astrocytes were more abundant compared to the other CA areas. In the GCL, astroglial cells were mainly located at the border towards CA4.

**Fig 8:**
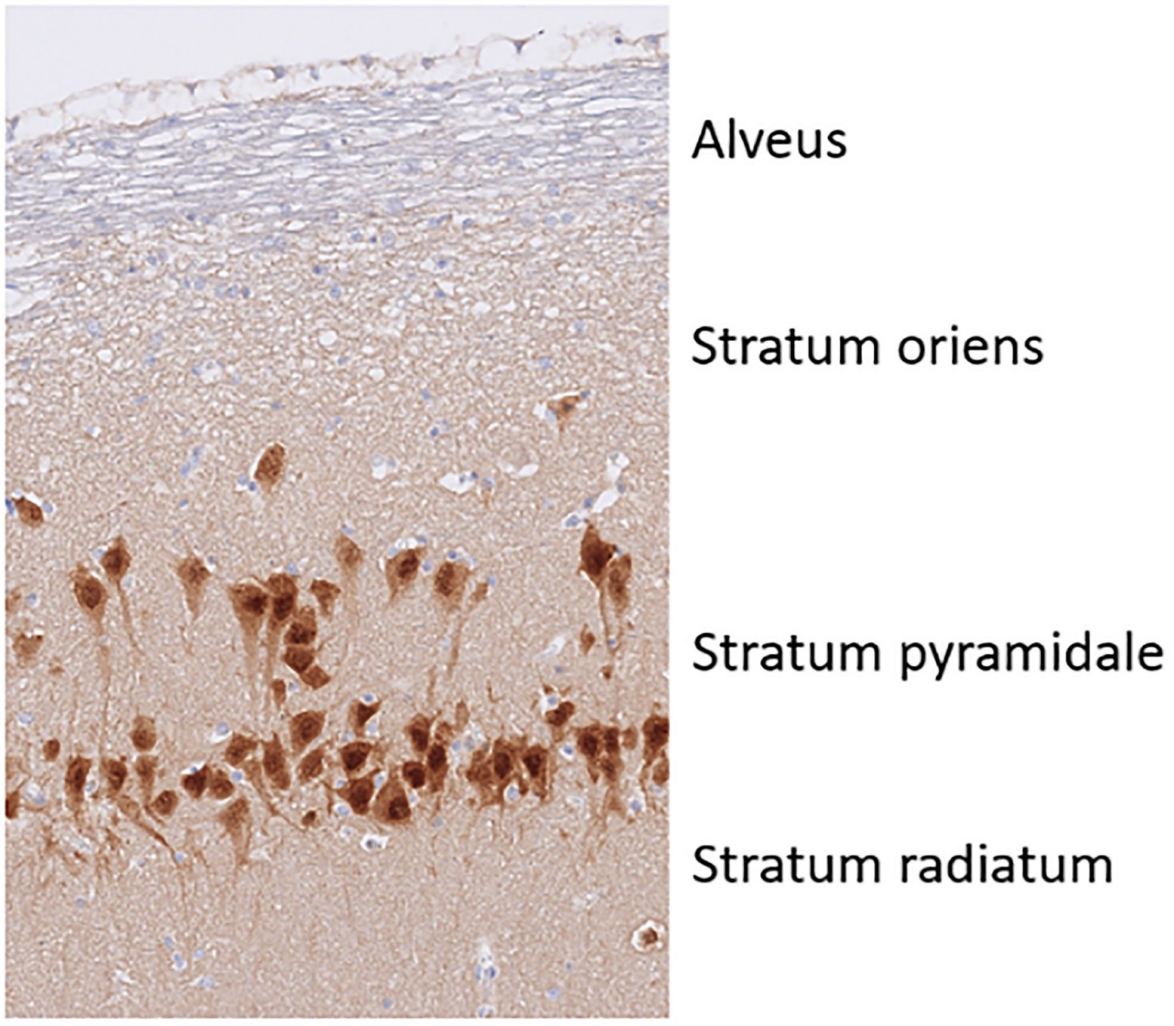
Schema of the hippocampal layers. The picture was extracted from the CA2 area of the hippocampal body of cat 17. In contrast to the six-layered isocortex of the forebrain, the architecture of the hippocampus and dentate gyrus is relatively simple. The principal neuron type in the hippocampus is the pyramidal cell and therefore the correspondent layer is called pyramidal cell layer or stratum pyramidale. In this layer, the perikarya of these cellular elements are located. The layer underneath the pyramidal cell layer is called the oriens layer (stratum oriens), which contains unmyelinated basal dendrites of the pyramidal cells. The layer that borders the ventricular surface of the hippocampus is the alveus. It is composed by the myelinated axons of the pyramidal cells. Directly above the pyramidal cell bodies the straight apical dendrites of the pyramidal cells can be seen in almost parallel orientation. This layer is called the radiant layer or stratum radiatum.

**Fig 9:**
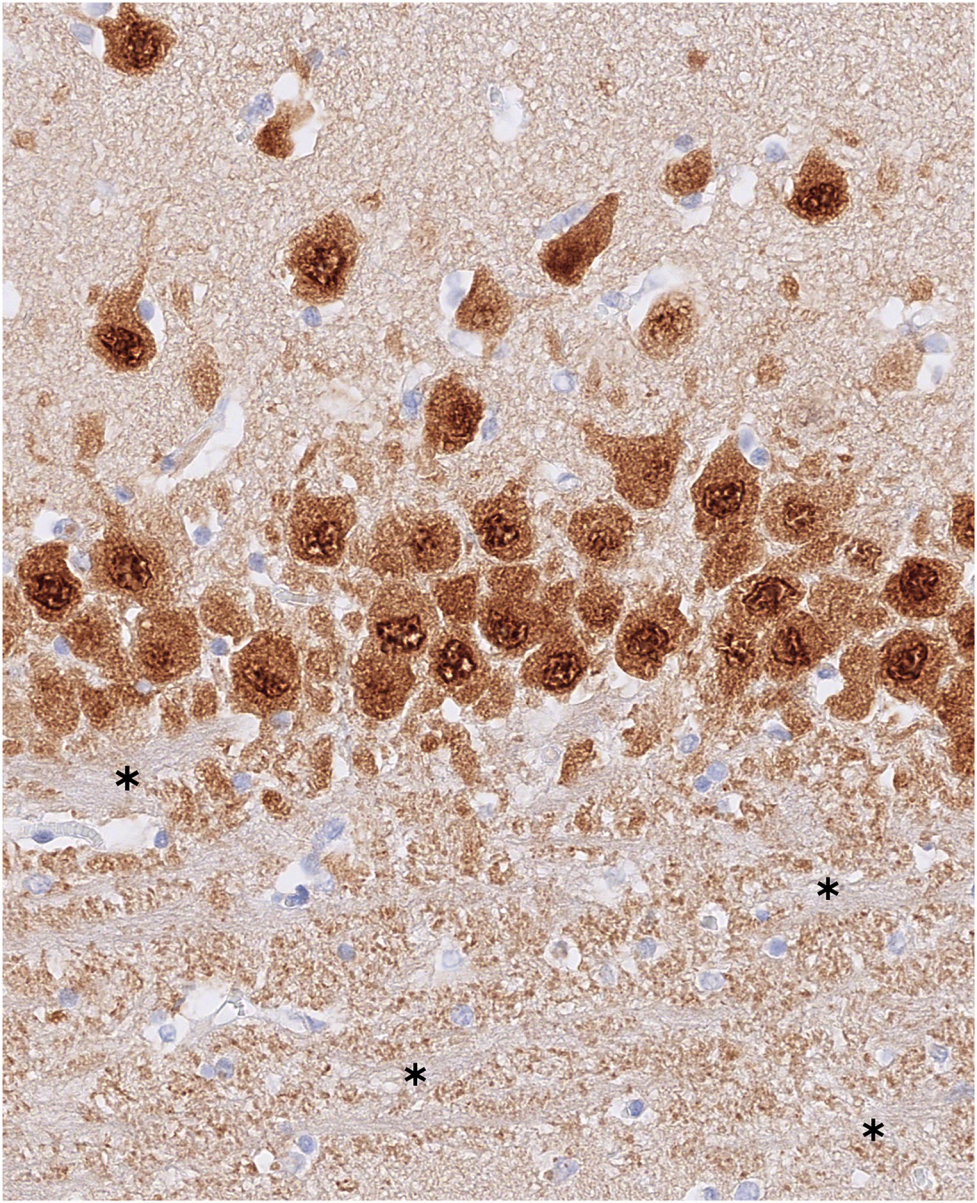
In this NeuN-stained slide from the hippocampal body of cat 17, the CA3 area with the underlying stratum lucidum can be seen. In this layer, the mossy fibers, which are the axons of the granule cells, are visible (asterisk).

Analysing the morphology of the examined areas (NeuN and GFAP), a marked difference in cytoarchitecture was observed (Fig 7). The pyramidal cell layer was divided, as in other species (i.e. mouse, human), into a superficial (closer to the stratum radiatum) and deep layer (closer to the stratum oriens) (Fig 8). This division was evident in CA1 and CA2, whereas it disappeared along CA3 and was completely absent in CA4. CA1 was thicker than CA2, CA2-3 dorsal and body, CA3 dorsal and body and the GCL in all animals. CA2-3 ventral (16.7% of the animals) and CA3 ventral (38.9%) showed LTs that were higher than in the corresponding section of CA1. In this area, the neurons presented an elongated to roundish form and were smaller than in the other CA areas. CA2 was a small transitional area whose extension differed along the hippocampal axis and from individuum to individuum as well. Its borders were not always easy to identify. In this area, the superficial layer was usually very thin and dense with polygonal to round pyramidal cells, whereas in the deep layer, neurons were sparser. Overall, CA2 was thinner than CA1 and usually also thinner than CA3. Indeed, only in one cat was CA2 thicker than CA3 (body). CA3 neurons were also roundish to polygonal cells. This neuronal population usually showed dispersion to some degree at the border with CA4. Beneath the pyramidal layer in the CA3 area, mossy fibres coming from the granule cells of the dentate gyrus could be consistently observed in the stratum lucidum (Fig 9). These structures and the related layer tended to disappear at the border with CA2.

In CA3, the distinction between deep and superficial layers was less evident, above all in proximity to CA4, which is the area in contact with the dentate gyrus. Here, the neurons had a rather polygonal form, were generally small and very dispersed, and showed as a very cell-poor area in the NeuN staining. Indeed, the AD in this layer was much higher than the ND (Fig 7).

Finally, the granule cells in the GCL of the dentate gyrus were very small, round neurons. Overall, this layer was very thin and compact, showing the highest neuronal densities. It did not reveal any division, as shown in the hippocampal CA areas. Here, the astrocytes were mostly located at the bottom of the layer, i.e. at the conjunction between the hilus (CA4) and DG, whereas in the hippocampal pyramidal cell layer, the astroglia was homogenously sparse throughout the full thickness of the stratum (Fig 7).

### Clinical history and quantitative histopathological examination of a cat with suspected idiopathic epilepsy

**Fig 10:**
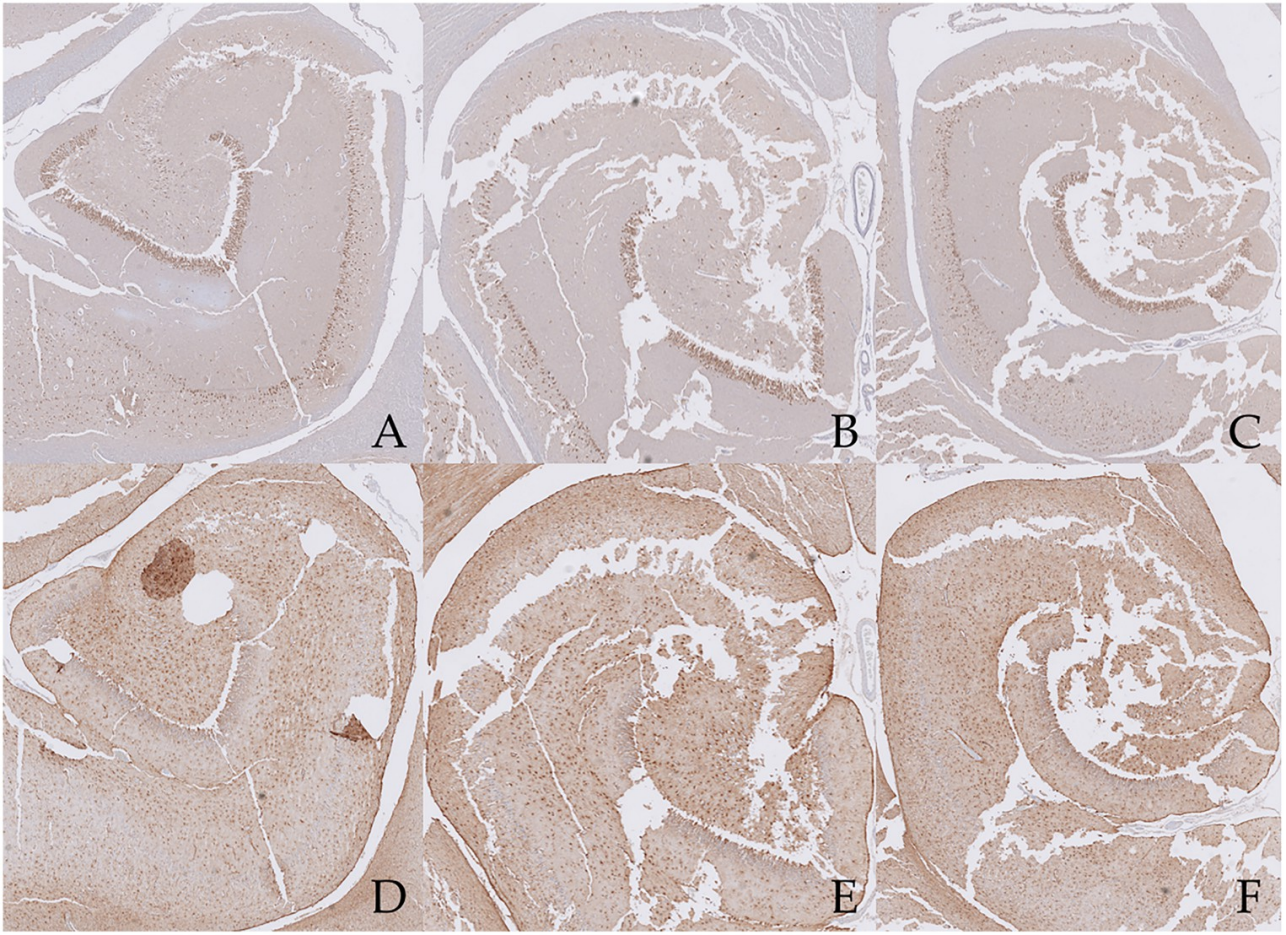
Scans of the NeuN- stained (A-C) and GFAP-stained (D-F) hippocampal sections from the epileptic cat. On the left the tail (A,D) of the hippocampus is visible, in the middle the body (B,E), and on the right the head (C,F). In all specimens, the CA3 area is the most damaged. Here, as well as in CA4, neuronal cells are partly degenerated and indeed, the NeuN immunostaining appears very pale in this area. Subjectively assessed, a mild neuronal loss and degeneration can be seen in the GCL. No further abnormalities are present in the dentate gyrus. In all three sections, a marked, generalized, anisomorphic astrogliosis is evident.

The histopathological examination of the brain of this cat evidenced only hippocampal changes. The stained sections from the hippocampal samples were markedly damaged and presented multiple lacerations and fissures (Fig 10), which were not observed on the specimens from normal brains. Due to excessive damage, it was not possible to perform two repeated measurements in all areas (S4 File). The examination of ND, AD and LT evidenced the presence of a mild to moderate decrease in ND (47.71–60.33% lower than the mean values for normal ND; see Table 2) and increase in LT (19.77–88% higher than the mean values for normal LT; see Table 4) at the level of the GCL in all three sections (tail, body and head) and of all CA areas but CA1 at the level of the tail. Except at the level of the GCL, in which a mild neuronal loss can be suspected (Fig 10A and 10B), these changes could not be appreciated visually without reference. Moreover, an increase in AD (22.33–122% higher than the mean values for normal AD; see Table 3) above all at the level of the hippocampal body and head, which was also subjectively evident (astrogliosis), was noted (Fig 10D–10F). Based on the reference values obtained in this study, this cat was diagnosed with a mild form of hippocampal sclerosis.

No statistical analysis was performed on these data.

## Discussion

Feline hippocampal sclerosis continues to be of great interest to researchers in veterinary epileptology but so far, little is known about this pathology in cats. Pathological studies have defined different patterns of hippocampal sclerosis, as well as its clinical course and the presence of associated brain diseases in affected cats [6]. Partial cortico-hippocampectomy is an emerging surgical technique in veterinary medicine, which will not only provide hippocampal specimens for histopathological investigations, but will also for conclusive correlations to be made between clinical signs, disease course, imaging and histopathology. Furthermore, these correlations will secondarily aid in advancing and refining the identification of suitable candidates for surgery [12, 13]. Lastly, feline hippocampal sclerosis might also offer a model to study human temporal lobe epilepsy [16].

In the current study, a further step in the diagnostic process of HS has been made. In fact, considering the possibility of performing surgical treatment on these animals in the near future, important considerations regarding the normal cytoarchitecture of the hippocampal pyramidal cell layer and the GCL have been made. Indeed, it has now been demonstrated that the hippocampal pyramidal cell population as well as the thickness of the pyramidal cell layer and GCL differ throughout the longitudinal hippocampal axis independently from age and breed in healthy cats, which is an important consideration to take into account while performing the histopathological examination of hippocampal specimens. In fact, the diagnosis of HS in both humans and cats is mostly based on the recognition of pyramidal cell loss and astrogliosis in hippocampal specimens [6, 11]. In addition, normal reference values on neuronal and astrocyte cellular densities as well as LT for the different CA areas (CA1 to CA4) and for the GCL of the dentate gyrus were provided and compared between the dorsal (tail), middle (body) and ventral (head) sections of the hippocampus. Such an analysis aimed to support the standardized histopathological diagnosis of HS in cats with antiseizure drug(s)-resistant mesial temporal lobe epilepsy (MTLE). Overall, no such evaluation has been undertaken in cats so far. However, a similar investigation has been performed in mice and demonstrated differences mostly in CA1 and in the suprapyramidal blade of the dentate gyrus along the dorso-ventral hippocampal axis [17]. In this case, only the neuronal densities of glutamatergic principal neurons were estimated. The presence of significant cytoarchitectural differences along the longitudinal hippocampal axis is also supported by evidence that lesions in the hippocampal tail affect learning and memory in rats [18], while lesions in the hippocampal head reduces fear-related behavior but do not impair spatial learning in rats [19]. This functional differentiation along the dorso-ventral hippocampal axis is actually well-known in monkeys [20] and humans [21] as well. In addition, a systematic review of cellular densities in the mouse brain, including the hippocampus, has been recently published [22]. In human medicine, some older studies investigated this topic, mostly aiming to find relations between neuronal densities in the hippocampus and pathological conditions, age or other possible influencing factors [23–27].

Since the study aimed to define a standardized examination protocol for the diagnosis of HS in cats, the relevant hippocampal and dentate gyrus areas examined in human medicine classification of this pathology were taken into consideration [11]: CA1-4 and the GCL. Regarding the method used for cell counting and for measuring LTs, a purposely simple technique was developed, so that this could be also employed in the diagnostic process for the histopathological examination of surgical hippocampal specimens, if needed. The technique is similar to the one used by Shimada et al. in 1992 for evaluating astroglial cell densities in the mouse [28]. In order to ensure a more precise analysis and reduce any bias, two measurements were made for all examined areas. Nevertheless, the t-test evidenced a statistically significant difference between some repeated measurements. These were the ND data in CA3 dorsal and CA4 dorsal and body, the AD data from CA3 ventral, the LT values from CA3 dorsal and the data obtained from the comparison between Nissl and NeuN staining for the evaluation of LTs from the CA2-3 area. Since most differences involved CA3, it can be postulated that a higher intrinsic variability is present in this CA area, which lies at the border between two very diverse regions (CA2 and CA4). Another speculation that can be made is that the method used for cell counting as well as the one used for the evaluation of LTs in this study could be responsible for this variability and therefore may not be completely appropriate for this type of assessment. Nevertheless, the intention of the authors was to use a technique that can be easily reproduced for the analysis of pathologic samples from animals with suspected hippocampal pathology. Such a method, like that of the optical disector [17, 29–31], would have likely been more accurate but inadequate for standard laboratory analysis.

In contrast to a previous study [6] in which age, responsible for mild neuronal loss, displayed a significant impact on the parameter interneurononuclear distance (INND) throughout CA1 to CA3, here age and breed did not show any statistically significant influence on ND, AD or LT comparing the hippocampal sections in the tail, body and head. In general, it is possible that these data would acquire a statistical significance if the study population was larger or older. Indeed, another recent study investigating cats as possible natural animal model for Alzheimer’s disease demonstrated the presence of a certain degree of neuronal loss in the hippocampus of old cats [32]. In this study, cats with only cerebral Aβ deposits but no hippocampal neurofibrillary tangles (NFT) showed a very mild, statistically not significant decrease in NeuN-positive cells if compared to younger animals, whereas cats with both Aβ deposits and hippocampal NFT presented a significant neuronal loss compared to the other two groups. Nevertheless, the median age of these animals was 17.6 years and therefore our study population was likely too young to reveal such statistically significant changes. The median age of the senior cats’ group of the present study was in fact only 13.6 years. In human medicine, contrasting opinions can be found in the literature regarding the influence of age on hippocampal cellular densities. Indeed, whereas in one study a decrease in pyramidal cell density in people over 68 years was found [24], in a later investigation exactly the opposite results were obtained [25]. Nevertheless, the two studies used different cell counting techniques and are therefore not strictly comparable. Moreover, in the second study, they supposed that the apparent increase in ND with age could also be attributed to the fact that the whole hippocampal formation (grey and white matter) was analyzed. In fact, these two components of the central nervous system, and in particular the white matter, undergo senescent shrinkage. Indeed, in agreement with some older studies [24, 26], it is rather unlikely that the proliferation of neurons takes place in the brains of adult humans. Senescent shrinkage could also explain the apparent increase in AD in senior cats, which was observed in this study in the hippocampal tail.

With regards to the variance analysis, a statistically significant difference in ND was observed along the dorsoventral hippocampal axis above all between the head and tail, and the body and tail, whereas the body and head seemed to have a more similar neuronal population. This difference was evident between all three sections (1, 2 and 3) in the GCL and completely absent in CA1. CA4 constituted an exception, since in this area, no differences in ND were observed between the body and tail. Overall, ND was higher at the level of the tail in CA2-3 and CA3 if compared to the body and head. The opposite trend was visible in CA4, where ND values were larger in the hippocampal head. With regard to the GCL, the ND showed at this level a net growth in the ventro-dorsal direction along the whole hippocampal axis. On the contrary, the astroglia exhibited a rather uniform cellular population, since only in CA1 and in the GCL were statistically significant differences found. Here, in both cases, AD was higher at the level of the hippocampal head. This trend is opposite to the one shown by neurons at the level of the GCL; indeed, ND was higher in the hippocampal tail.

Regarding LTs, net differences were pointed out between the head and tail, as well as between the body and tail in all areas, whereas only CA3 and the combination of CA2-3 showed differences between the hippocampal head and body. The LTs tended to increase in the dorsoventral direction in the CA2, CA2-3 and CA3 areas. Instead, the opposite trend was observed in CA1 and in the GCL. Overall, this analysis suggests that, in the evaluation of hippocampal specimens, the hippocampal section (tail, body or head) to which the examined tissue sample belongs should be taken into consideration, since many differences were observed, above all in ND and LT.

With regard to the clinical relevance of this study, although the diagnosis of hippocampal sclerosis is normally reached without a proper assessment of cell densities in human medicine, it has to be considered that human patients, who are affected by this pathology, usually have a long clinical history of seizures before undergoing surgical treatment. Indeed, it has been reported in a large multicenter study that the average interval between the onset of epilepsy and surgical intervention is 24 years among epileptic people [33]. Furthermore, in another study, in which only patients with MTLE were investigated, the average period of time was 18 years [34]. Now, considering that most domestic cats, even if completely healthy, do not live so long, it can be postulated that some of the cats affected by HS would present border-line hippocampal changes, which are not so obvious as in affected human hippocampi. This discrepancy could lead to misinterpreting the histopathological findings from some animals affected by HS. For this reason, in those cases with subtle hippocampal changes, the quantitative evaluation of cellular densities and LT could be helpful to obtain a definitive diagnosis. Nevertheless, it must be taken into consideration that the assessment of cellular densities alone can be sometimes not an easily interpretable indicator of disease state, as differences can rely on both changes in cell number and cell distribution (i.e. presence of layer dispersion) [35] and therefore it is recommended to evaluate both ND and LT.

The recently published first case report of focal cortical resection and hippocampectomy in a cat with non-induced, refractory temporal and occipital seizures showed that the examination of such small and surgically deteriorated specimens may not be easy. Indeed, in this case, an accurate assessment of neuronal cell loss was not possible and therefore it could not be determined whether the cat was affected by typical HS [12]. This first example of a surgically treated cat as well as the epileptic cat examined as part of this study further confirmed the hypothesis that whereas substantial cellular loss like the one observed in hippocampal specimens from affected human patients does not require a quantitative examination to obtain a diagnosis of HS, on the other hand, in veterinary medicine, it can be supposed that a large number of patients would present only subtle evidence of neuronal loss. In addition, considering that resected specimens can undergo a certain degree of damage during surgery, this makes a subjective examination of resected tissues even more difficult. Therefore, we suggest that a quantitative analysis should be performed in dubious cases. In the epileptic cat examined here, the extensive damage to the tissues was probably of artefactual origin, due to delayed fixation in formaldehyde.

The choice of staining (Nissl, NeuN and GFAP) was also in agreement with the standard investigation protocol used in human medicine in patients with suspected HS [11]. However, it must be considered that the use of the anti-GFAP antibodies to identify non-reactive astrocytes may have led to an undercounting of the astroglial cell population [36]. Indeed, not all astrocytes express this marker in the cortex and hippocampus of adult animals [37–40]. For this reason, it cannot be excluded that the astrocyte cell count was underestimated in this study as well as the astroglial densities. In this regard, a comparative examination with the astroglial marker S100β should be performed to verify this hypothesis. In fact, in contrast to GFAP, it seems that all astrocytes express S100β, which is on the other hand less specific than GFAP. In fact, it can also be found in some types of neurons, at least in rats [41].

The main limitation of the study is related to the small population examined, which could of course have led to a bias. Moreover, the difficulties performing NeuN staining led to the consequence that ND was evaluated in an even smaller group of 14 animals. A hypothesis for the failure of the NeuN immunostaining in some cats is that this was the consequence of overfixation of the examined tissues in formaldehyde or delayed fixation of the tissues (even if within 12 hours of death), which led to a neuronal nuclear damage. Other explanations could not be found since all brains were processed in the same manner and stained using the same kits and staining system.

The hippocampus from only one epileptic cat was examined in this study to compare the normal reference values obtained from the statistical analysis with pathological ones. As a next step, a statistical analysis including an adequately large population of epileptic cats with suspected hippocampal pathology should be considered in order to assess relevant differences in comparison to normal cats and to further evaluate the clinical importance of a quantitative histopathological examination of the hippocampus in these animals.

## Conclusions

The present study introduces a guide for processing postmortem feline brains in order to perform a standardized morphological analysis of the hippocampal areas. The data provide for the first-time reference values for neuronal and astroglial densities as well as for the LT of the hippocampal pyramidal cell layer (CA1-4) and GCL in adult and senior cats. Normal values are crucial to estimate the hippocampal pathology. As surgical treatment (i.e., hippocampectomy and/or cortical resection) for epileptic cats with antiepileptic drug(s) resistance and seizures of temporal lobe origin may become a therapeutic option in the future, this study will help in the standardized histopathological examination of resected hippocampal specimens.

## Supporting information

S1 File ND repeated measurements from the NeuN stained hippocampi. For each evaluated area two measurements were performed in order to reduce any bias. The quantification of the ND was not possible in cats 5, 6 and 9 due to deficient of absent NeuN staining.
(XLSX)

S2 File AD repeated measurements from the GFAP stained hippocampi. For each evaluated area two measurements were performed in order to reduce any bias.
(XLSX)

S3 File LT repeated measurements from the Nissl and NeuN stained hippocampi. For each evaluated area two measurements were performed in order to reduce any bias. The quantification of the ND in the NeuN- stained slides was not possible in cats 5, 6 and 9 due to deficient of absent NeuN staining.
(XLSX)

S4 File ND, AD and LT repeated measurements from the NeuN-, GFAP- and Nissl-stained hippocampi. Due to extensive tissue damage, it was not possible to perform two measurements for each evaluated area and in few areas not even one.
(XLSX)
